# Composite Materials Based on Gelatin and Iron Oxide Nanoparticles for MRI Accuracy

**DOI:** 10.3390/ma15103479

**Published:** 2022-05-12

**Authors:** Mioara Drobota, Stelian Vlad, Luiza Madalina Gradinaru, Alexandra Bargan, Iulian Radu, Maria Butnaru, Cristina Mihaela Rîmbu, Romeo Cristian Ciobanu, Magdalena Aflori

**Affiliations:** 1 “Petru Poni” Institute of Macromolecular Chemistry, Aleea Gr. GhicaVoda, 41A, 700487 Iasi, Romania; vladus@icmpp.ro (S.V.); gradinaru.luiza@icmpp.ro (L.M.G.); anistor@icmpp.ro (A.B.); mariabutnaru@yahoo.com (M.B.); 2 Department of Surgery, Regional Institute of Oncology, I-st Surgical Oncology, “Grigore T. Popa” University of Medicine and Pharmacy, 700483 Iasi, Romania; raduiuli@gmail.com; 3 Department of Biomedical Sciences, “Grigore T. Popa” University of Medicine and Pharmacy, Kogalniceanu Street, 9-13, 700115 Iasi, Romania; 4 Department of Public Health, Faculty of Veterinary Medicine, “Ion Ionescu de la Brad” University of Life Sciences, Mihail Sadoveanu Alley no. 8, 700490 Iasi, Romania; crimbu@uaiasi.ro; 5 SC All Green SRL, I. Bacalu Street, 5, 700029 Iasi, Romania; rciobanu@yahoo.com; 6 Electrical Engineering Faculty, “Gheorghe Asachi” Technical University of Iasi, Dimitrie Mangeron Bd., 67, 700050 Iasi, Romania

**Keywords:** gelatin, iron oxide, tensile properties, dynamic vapor sorption analysis, magnetic determination, antibacterial activity

## Abstract

The majority of recent studies have focused on obtaining MRI materials for internal use. However, this study focuses on a straightforward method for preparing gelatin-based materials with iron oxide nanoparticles (G–Fe_2_O_3_ and G–Fe_3_O_4_) for external use. The newly obtained materials must be precisely tuned to match the requirements and usage situation because they will be in close touch with human/animal skin. The biocompatible structures formed by gelatin, tannic acid, and iron oxide nanoparticles were investigated by using FTIR spectroscopy, SEM-EDAX analysis, and contact angle methods. The physico-chemical properties were obtained by using mechanical investigations, dynamic vapor sorption analysis, and bulk magnetic determination. The size and shape of iron oxide nanoparticles dictates the magnetic behavior of the gelatin-based samples. The magnetization curves revealed a typical S-shaped superparamagnetic behavior which is evidence of improved MRI image accuracy. In addition, the MTT assay was used to demonstrate the non-toxicity of the samples, and the antibacterial test confirmed satisfactory findings for all G-based materials.

## 1. Introduction

Novel materials such as polymeric hydrogels [1,2], carbohydrate polymers [3,4,5,6], lipids [7,8], inorganic carriers [9], and bio-macromolecular scaffolds [10,11] coupled with various particles were obtained for different biomedical applications.

A biomaterial is a material that has been engineered to interact with biological systems for a medical scope, either a therapeutic or a diagnostic one. Over the centuries, biomaterial development has presented a growing interest to researchers of both scientific and technological applications. Recently, polyelectrolyte gels have received much attention due to their properties. Polyampholytes materials containing both cationic and anionic groups on their macromolecule chains have also been intensively studied [12,13]. Some natural polymers, such as proteins, are also polyampholytes, and their behavior was tested in different applications [14,15]. Gelatin is a high-molecular-weight polypeptide derived from collagen with biocompatible behavior, and high bioadhesivity [16]. Gelatin is a natural biopolymer with relatively low production cost and interesting functional properties [17,18].

The organic or inorganic nanoparticles widely used in the polymer matrix, (e.g., silica [19,20], gold [21], or silver [22] by physical or chemical interactions, lead to the preparation of hybrid materials with novel properties that are very useful in biomedical applications. These materials can be obtained by introducing magnetic nanoparticles, mostly magnetite [23], maghemite [24], or cobalt ferrite (CoFe_2_O_4_) [25] into the polymer matrix. The newly obtained hybrid materials are designed to increase clarity and visibility through contrast agents by using nanoparticles such as TiO_2_ (titanium dioxide) or AuNP (gold nanoparticles) [26]. Magnetic nanoparticles (MNP) are often used due to their unique features [27], being responsive to external magnetic fields, and they are intensively studied as imaging MRI contrast agents.

Magnetic nanomaterials like iron oxide (Fe_2_O_3_ and Fe_3_O_4_) are transported directly to the target with no adverse effects on the body [28] for applications in different areas such as target drug delivery, tumor and cancer diagnosis and treatment [29,30,31,32], and magnetic resonance imaging (MRI) [33].

Many researchers studied gelatin-coated Fe_3_O_4_ nanoparticles [34], and others were working on gelatin grafted with Fe_3_O_4_ for applications in combined chemotherapy and hyperthermia. Due to the structure of gelatin molecules on the surface of MNPs, these materials present a hydrophilic behavior and provide sites for the encapsulation of anticancer drugs through hydrophobic interaction [35,36].

It is well known that nanoscaled magnetic particles have attracted much attention because magnetic nanoparticles have shown great potential for applications in the biomedical field. MRI has become an important diagnostic tool as a noninvasive technique routinely used in clinics for disease diagnosis in hospitals. Due to the development of nanotechnology, new nanoparticle MRI were obtained as contrast agents with further improved imaging abilities as well as other functions [37]. Many approaches have been focused on the encapsulation of magnetic nanoparticles with polymeric materials, such as dextran, chitosan, gelatin, poly(ethylene glycol) (PEG), PLGA, poly(d, l-lactide) (PLA), and poly(glycolide) (PGA), etc. because these materials have biocompatible and biodegradable properties as well as low toxicity [38]. Iron oxide was used as a superparamagnetic contrast agent in two forms: superparamagnetic iron oxide (SPIO) and ultrasmall superparamagnetic iron oxide (USPIO) with successful outcomes in the diagnosis of tumors. The aim of this study was to develop a gelatin-based material with superparamagnetic iron oxide nanoparticles as a potential device in MRI applications. Indeed, the oral or injectable contrast agents have been developed, but only a few works develop external contrast agents by using the NMR method, which is a hazardous objective. We try to obtain some external device with an amplifier role of MRI images [39,40]. In the presence of an external magnetic field when radiofrequency (RF) pulses with Larmor frequency, the nuclei become excited and transit between low and high energy levels. There are some parameters which must be met, such as the requirements that the magnetic particles must have a high saturation magnetization, so the dipolar interactions become very small when the particle size becomes very small. This will serve to minimize particle aggregation when the field is applied [41].

The aim of our study is to develop novel composite gelatin-based materials with iron oxide magnetic nanoparticles (Fe_2_O_3_ and Fe_3_O_4_ as the nano-sized fillers) in order to improve the image quality in MRI investigations for external use. The good clarity of MRI images can be provided by gelatin (a biopolymer with excellent optical properties) [42,43] and by iron oxide nanoparticles (which assure signal amplification) [44].

Therefore gelatin-based materials were prepared via solution casting by embedding different amounts of Fe_2_O_3_ and Fe_3_O_4_ nanoparticle into a gelatin matrix. The structural, morphological, wettability, magnetic, mechanical and dynamic vapors’ sorption properties were investigated. The cytotoxicity (controlled by MTT assay) and the antibacterial tests were also performed.

## 2. Materials and Methods

### 2.1. Materials

Gelatin from porcine skin, type A, tannic acid, sodium hydroxide (NaOH) ACS reagent, ≥97.0%, pellets, were purchased from Sigma Aldrich (Steinheim, Germany). Bidistillate water was freshly prepared prior to use. Iron (III) oxide nano powder (Fe_2_O_3_) of <50 nm nanoparticles (Nps) size and Iron (II, III) oxide nano powder (Fe_3_O_4_) of 50–100 nm Nps size (Scanning Electron Micoscopy, SEM) were used as the magnetic particles throughout the investigation and were also purchased from Sigma-Aldrich (Steinheim, Germany).

### 2.2. Preparation Methods

#### 2.2.1. Preparation of Gelatin-Based Materials

**Figure 1:**
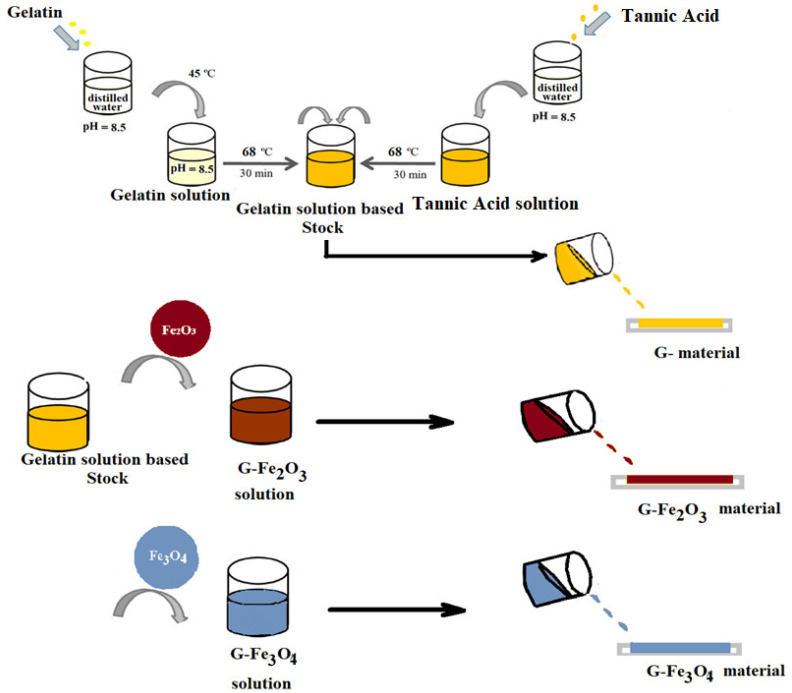
Scheme for obtaining gelatin-based materials embedded with iron oxide nanoparticles.

In the present study, the gelatin-based solution with a concentration of 10% (wt) was obtained, from gelatin dissolved in distilled water at a final pH = 8.5. Prior to the preparation of the gelatin-based solution pH was stabilized by using a necessary quantity of 4M NaOH solution. The gelatin solution was continuously stirred until the solution appeared homogeneous, and then the temperature was raised to 45 °C and stirred for another 2 h. Then, the temperature was raised to 68 °C, and a solution of tannic acid was added. The reaction occurs under continuous stirring for 30 min. Separately, tannic acid solution was obtained in distilled water at the same pH = 8.5 [45]. The concentration of gelatin-based materials is 40 mg/g (tannic acid/gelatin) (Figure 1). When the reaction between gelatin and tannic acid was complete, the solution became clear.

#### 2.2.2. Preparation of G, G–Fe_2_O_3_ and G–Fe_3_O_4_ Materials

One part of stock solution was used to obtain a film of gelatin noted with a G code (Figure 1). The second part was separated into two equal solutions parts. In both solutions we introduced 0.05% (wt) Fe_2_O_3_ and Fe_3_O_4_ nanoparticles, respectively, and the obtained films were denoted as G–Fe_2_O_3_ and G–Fe_3_O_4_. The homogeneity of magnetic solutions was obtained by ultrasonication for 5 min. After that, all the films were obtained by casting on a Teflon support and then drying in the oven for 48 h, in controlled conditions (temperature 24 °C, low pressure (1 Hg). The thickness of the prepared composite films was around 0.2 mm, measured with a digital micrometer.

**Figure 2:**
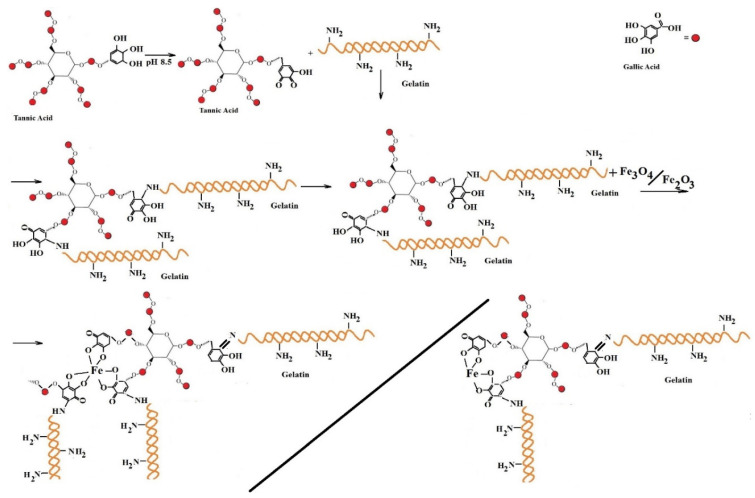
Illustration of possible interactions between tannic acid, gelatin, and iron oxide nanoparticles.

Figure 2 presents possible interactions between the molecules of gelatin, tannic acid, and iron oxide. Gelatin interacts with tannic acid, which acts as a crosslinking agent, most often to form the intra- and intermolecular hydrogen bonds and sometimes to form covalent bonds like Schiff bases. The OH groups of tannic acid interact with the amino functional groups of the amino acid units in gelatin at an alkaline pH of 8.5. Some amino functional groups of amino acid units in gelatin (such as lysine, arginine, and histidine) could react with phenolic reactive sites of TA under alkaline conditions to form covalent C–N bonds and generate cross-linking [46,47]. On the other hand, the phenolic hydroxyl groups of TA could be associated with gelatin chains via hydrogen bonding. At a pH higher than the isoelectric point (pI = 9), the gelatin macromolecules are negatively charged, as explained by the loss of protons from amino and carboxyl groups [48]. Water molecules in gelatin hydrogels are replaced by iron ions and strong ion crosslinks are formed between molecular chains by the strong action [49].

### 2.3. Characterization Techniques

#### 2.3.1. Fourier Transform Infrared Spectroscopy

The spectra for all the films were recorded with a Fourier-transform infrared with attenuated total reflectance (ATR-FTIR) spectrometer, and the spectra were recorded from an average of 64 scans by using a Bruker LUMOS-FTIR microscope. (Bruker Optik GmbH, Ettlingen, Germany). The ATR reflection module (Attenuated Total Reflection, Ettlingen, Germany) was equipped with a diamond crystal and a single reflection at a 45° angle and OPUS 8 software for spectral processing. The spectra were collected at a wavenumber range of 4000–500 cm^−1^. The iron oxide nanoparticle samples were pelletized with KBr and recorded by using the transmission module, and the spectra in this mode were collected between 4000–400 cm^−1^ wavenumbers. Air spectrum was used as background correction. All spectra were monitored with a resolution of 4 cm^−1^.

#### 2.3.2. Contact Angle and Surface Wettability Study

Static contact angle (SCA) measurements were monitored via the sessile drop method by using a CAM 101 Optical Contact Angle Instrument (KSV Instruments, Helsinki, Finland) containing a special optical device (charge-couple device (CCD)) related to a computer. Surface tension parameters of the samples were evaluated by using the method developed by Owens, Wendt, Rabel, and Kaelble [50], based on the SCA values. For each kind of liquid, five different regions of the surface were selected to obtain a statistical result. For these studies, solvents included double-distilled water and ethylene glycol.

#### 2.3.3. Scanning Electron Microscopy (SEM) Analysis-EDAX

Surface morphology, dispersion of iron oxide nanoparticles in gelatin-based materials, and elemental composition were evaluated with a Verios G4 UC scanning electron microscope (Thermo Scientific, Waltham, MA, USA) equipped with an X-ray spectroscopy analyzer with energy dissipation (Octane Elect Super) and an SDD Detector (AMETEK, Tokyo, Japan). Prior to image acquisition, the samples were coated with 6 nm platinum by using a Leica EM ACE 200 Sputter Coater (Leica Microsystems, Vienna, Austria) to ensure optimum electrical conductivity and to prevent accumulation of charge during exposure to the electron beam. SEM investigations were performed in high vacuum mode by using a secondary electron detector (Everhart—Thornley Detector, ETD, The Netherlands Company, Brno, produced in Brno, Czech Republic) at an acceleration voltage of 5 kV.

#### 2.3.4. Optical Properties

Reflectance analysis of the gelatin-based samples was performed on a Lovibond LC 100 instrument (Tintometer Ltd., Wiltshire, UK). The CIELAB standard systems (L*a*b*) that present the colors in relation to a system of Cartesian axes has been used in countless studies to determine color measurements, but these measurements are not relevant in our study. The equipment was used to determine the opacity parameter, a procedure that was detailed in the paper of Barzic et al. [51].

#### 2.3.5. Determination of Dynamic Vapors’ Sorption and Diffusion Coefficients

The dynamic vapors’ sorption (DVS) capacity of the gelatin-based materials samples was measured in a dynamic regime by using an automated gravimetric analyzer IGAsorp device (Hiden Analytical, Warrington, UK). The IGAsorp is a standard sorption apparatus, equipped with a sensitive microbalance (resolution 1 mg and capacity 200 mg), which continuously registers the weight of the sample at the same time with the temperature and relative humidity in the sample chamber. Based on the experimental isotherm data of the sorption–desorption experiment at 25 °C, the diffusion coefficients could be determined by using Fick’s first and second laws. Crank [52] deduced that the diffusion coefficient for short time periods (*M_t_*/*M_∞_* < 0.5) is described by Equation (1):

(1) $$\frac{M_{t}}{M_{\infty }}=\frac{4}{l}\sqrt{\frac{D_{1}\cdot t}{\pi }}$$

where *M_t_* (g) is the mass of sorbed water vapor at time *t* (s), *M_∞_* (g) is the mass sorbed at *t* = ∞, *l* (cm) is the sample thickness and *D* (cm^2^/s) is the Fickian diffusion coefficient. The Fickian diffusion coefficient of the water vapor was determined from the initial slope of the (*M_t_*/*M_∞_)*^2^ versus *t*^1/2^ plot (extracted from kinetic sorption experiments). Over a longer time (*M_t_*/*M_∞_* > 0.5), the Fickian diffusion coefficient could be deduced from Equation (2):

(2) $$\frac{M_{t}}{M_{\infty }}=1-\frac{8}{\pi^{2}}\cdot e^{\frac{-D_{2}\cdot \pi^{2}\cdot t}{l^{2}}}.$$

*D*_1_ is the diffusion coefficient calculated for short time periods (*M_t_*/*M_∞_* < 0.5). *D_2_* is the diffusion coefficient for long time periods (*M_t_/M*_∞_ > 0.5) where (*M_t_*/*M_∞_t*^2^ = 16·*D*_1_·*t/π·l*^2^ = K_1_·*t*, so that: K_1_* = 16·*D*_1_/*π·l*^2^ resulting in *D*_1_ = K_1_*πl*^2^/16 and ln(1 − *M_t_/M*_∞_) = ln8/*π*^2^ − *D*_2_·*π*^2^*·t*/*l*^2^ = K_2_*·t*, where: K_2_* = −*D*_2_·*π*^2^/*l*^2^, resulting in *D*_2_ = −K_2_*l*^2^/*π*^2^.

#### 2.3.6. Determination of Tensile Properties

Tensile tests of the gelatin-based materials strips (50 mm × 8.5 mm × 4 mm) were carried out at room temperature by using a universal testing machine (Instron, Norwood, MA, USA), operated with a speed of 30 mm/min. Five replicates were used for each sample to obtain the averaged values and standard deviation. Elongation at break and tensile strength values were directly determined from the stress–strain curves. The Young’s modulus was evaluated from the slope of the stress–strain curve in the linear region at low deformation.

#### 2.3.7. Magnetic Determination

The assessment of the magnetic properties of the samples was performed at ambient temperature by using an MPMS3 (7 T) SQUID vibrating-sample magnetometer (VSM) operated in DC mode (Lake Shore Cryotronics, Woburn, MA, USA). The magnetic field was swept from −30,000 to 30,000 Oe with a stepwise progression of 50 Oe.

#### 2.3.8. Biological Test

##### Cytocompatibility—MTT Assay

The cytocompatible characteristic of the gelatin based-material was assessed by the MTT (i.e., 3-(4,5-dimethyltiazol-2-yl)-2,5-diphenyl tetrazolium bromide) colorimetric assay [53,54,55], through which the metabolic activity of the live cells in the presence of the G-extracts is evaluated. The extracts of G-materials are obtained in the cultures by using 24-well Greiner Bio-One hanging cell culture inserts with PET membrane with a pore size of 0.4 µm. In this respect, G-material with 4 mm in diameter were placed into inserts, over the monolayer of rabbit primary dermal fibroblasts, and incubated up to 48 h. After specified periods (i.e., 1 and 2 days) of the continuous exposition of the cells to 100% gelatin-based extracts, the inserts were taken out from the wells (12), and the cells were quantified concerning their viability profile.

For this objective, the cells were washed with phosphate-buffered saline (PBS), treated with MTT solution (5 mg/mL) diluted in fresh medium, and kept at 37 °C for 3 h in order to provide the formazan crystal formation (as a dark-blue insoluble product) [53,54,55]. The formazan crystals were solubilized with isopropyl alcohol under orbital agitation at 150 rpm by using an ES-20 benchtop shaker–incubator (Environmental Shaker-Incubator ES-20, Biosan, Riga, Latvia), for 15 min., and the absorbance was read by using a microplate reader (Tecan Sunrise, with Magellan V.7.1 soft, Tecan Group Ltd., Männedorf, Switzerland) at a wavelength of 570 nm, of the resulting extract from each well.

##### Antibacterial Test

An antimicrobial activity test of the films was carried out by using the agar diffusion method described by Maizura et al. [56]. The antibacterial effects of the films were determined by the inhibition zone against *Staphylococcus aureus* (*S. aureus*) and *Escherichia coli* (*E. coli*) on solid media. The antibacterial activity has been performed by using the Kirby–Bauer Disk Diffusion Method [57], adequate for testing gelatin material-based samples.

The composites were cut in the form of 6-mm discs and were put in contact with two reference bacterial strains: *S. aureus* ATCC 25,923 (Gram positive) and *E. coli* ATCC 25,922 (Gram negative). The solutions were prepared by bacterial inoculation for 24 h of cultured cells, with a 0.9% NaCl dilution and a turbidity of 0.5 by using the McFarland scale (1.5 × 108 bacterial cells/mL). The bacterial culture has been incorporated into the culture medium Muller Hinton Agar (Oxoid) melted and cooled to 45 °C. The samples were tested on the surface of the culture medium at a position relatively equal to one another. After that, the plates were brought in standard conditions at 37 °C for 24 h. The estimation of the antimicrobial effect was made qualitatively and semi-quantitatively by measuring the area of microbial inhibition, created around each tested sample [57].

## 3. Results and Discussion

### 3.1. FTIR and ATR-FTIR Analysis

**Figure 3:**
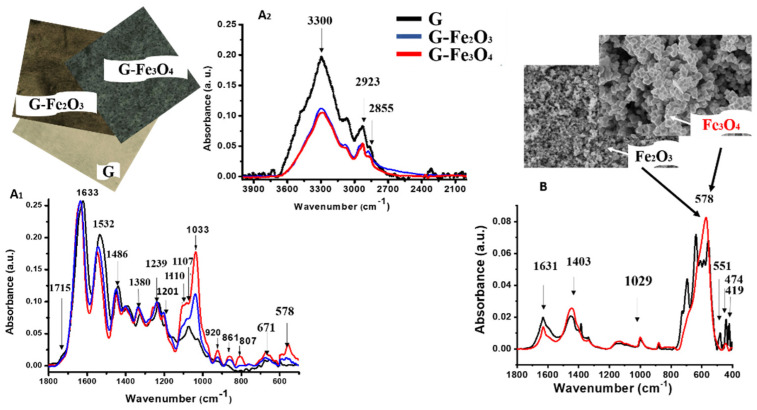
ATR-FTIR spectra of gelatin-based materials (G, G–Fe_2_O_3_ and G–Fe_3_O_4_) in the range: 4000–2100 cm^−1^ (**A1**) and 1800–500 cm^−1^ (**A2**); (**B**) FTIR spectra of pure Fe_2_O_3_ and Fe_3_O_4_ nanoparticles in the range: 1800–500 cm^−1^.

Infrared spectroscopy is an important and common investigative technique, which analyzes information about the molecular chemical structure and provides the relationship between structure and properties. The gelatin-based films were investigated by ATR-FTIR and corresponding spectra are illustrated in Figure 3 (A1 and A2). ATR-FTIR spectra for gelatin-based films with and without Fe_2_O_3_ and Fe_3_O_4_ nanoparticles are shown in Figure 3A. In the range 3400–3100 cm^−1^, the characteristic (ν-stretching vibrations) νNH and νOH vibrations are attributed to amide A with a central peak at 3300 cm^−1^. The position of the absorption band confirm that phenolic hydroxyl groups are implied in the gelatin/polyphenol reaction from tannic acid [58,59]. The vibration bands at 2923 and 2855 cm^−1^, respectively, were assigned to ((νs) symmetric and (νas) asymmetric stretching vibrations) νsCH_2_ and νasCH_2_.

The peaks from the ATR-FTIR spectrum of G material could be assigned as follows: amide A (3400–3350 cm^−1^, attributed to -νNH and -νOH vibrations), amide B (3085–3070 cm^−1^), amide I (1700–1600 cm^−1^, corresponding to predominantly νC = O vibrations of protein amide), amide II (1600–1500 cm^−1^, assigned to 60% bending (δ) vibrations NH and 40% of νC-N vibrations) and amide III (1200–1300 cm^−1^, corresponding to νC-N and δN-H; this vibration overlaps with—δCH_2_ vibrations). The vibration that appeared at 1715 cm^−1^ indicates the presence νCO of carbonyl group of tannic acid from G-material [60].

The presence of amide I has a major contribution to the specific band of ν(C = O) from the strong absorption band at 1633 cm^−1^. The amide II located in spectrum at 1532 cm^−1^ is a complex band and is formed mainly from the vibrations of the δ(NH) and ν(CN) groups belonging to the amino acid molecules of the protein composition. The signal at 1380 cm^−1^ was assigned to the symmetrical band δ(CH_3_) and the peak at 1239 cm^−1^ was assigned to δ(CH_2_). The vibration ν(CN) from amide III was founded at 1201 cm^−1^ and can be attributed to tyrosine and phenylalanine (molecular chain).

In Figure 3B the FTIR spectra of Fe_2_O_3_ and Fe_3_O_4_ nanoparticles are presented. Both samples were investigated in the range 4000–400 cm^−1^. The interval between 400 and 700 cm^−1^ presents the characteristic bands of Fe_2_O_3_ nanoparticles (for Fe_3_O_4_, a single characteristic peak was observed in this interval) [61,62,63]. The absorptions between 400–600 cm^−1^ from spectrum of Fe_2_O_3_ Nps which have a different size, were attributed to the stretching vibration of Fe–O symmetric stretching [62,63], a small peak at 1029 cm^−1^ was assigned to Fe–O in bending vibration [64,65,66] and the band at ca. 1400 cm^−1^ which is probably due to carbonate on iron particles [67] or adsorbed water on the surface of iron oxide nanoparticles [68,69]. The vibration at 1631 cm^−1^ is characteristic for both Fe_2_O_3_ and Fe_3_O_4_ nanoparticles [70,71]. The FTIR spectrum of Fe_3_O_4_, Figure 3B showed a strong vibration at 578 cm^−1^, indicating the presence of characteristics vibrations νFe–O of the Fe_3_O_4_ Nps [63].

The main bands of G, G–Fe_2_O_3_ and G–Fe_3_O_4_ materials are present on the spectra. The vibrations ν (C–O) attributed to the carboxylic acid and ether strip at 1107 and 1028 cm^−1^ of the tannic acid present in the material G are more intense and shifted to 1110 and 1033 cm^−1^ in the case of G–Fe_2_O_3_ and G–Fe_3_O_4_ composites. These changes occurred as results of the formation of Fe–O chelates [72,73]. The ATR-FTIR spectrum of G–Fe_3_O_4_ material showed a little shoulder which appears at 1107 cm^−1^, and which could be attributed to the νO-H vibration in interaction with Fe_3_O_4_ nanoparticles, confirming the interaction between gelatin and Fe_3_O_4_ nanoparticles.

It was observed that an absorption increase at 1201 cm^−1^ for G–Fe_3_O_4_ and G–Fe_2_O_3_ compared to G material, due to the presence of the adjacent hydroxyl phenolic groups, (from tannic acid groups) in the composite material which will increase the stability of iron complex ions [72,73,74]. The peaks around 1380 and 1485 cm^−1^ belong to Fe^3+^ or Fe^2+^ complex ring vibrations proving the chelates formation [74]. The vibrations at 1110 cm^−1^ are assigned to the δFe–O–H groups of gelatin, due the presence of νC-OC groups from the β-glycosidic rings in interaction with Fe_3_O_4_ [72]

Moreover, in the ATR-FTIR spectrum of G–Fe_2_O_3_, the presence of the band at 853 cm^−1^ associated to the Fe–O of Fe_2_O_3_ [75,76] is observed. The vibrations at 920, 861, 807, and 671 cm^−1^ are also characteristic bands of Fe_3_O4 nanoparticles [70,76].

### 3.2. Surface Morphology and Hydrophobicity

**Figure 4:**
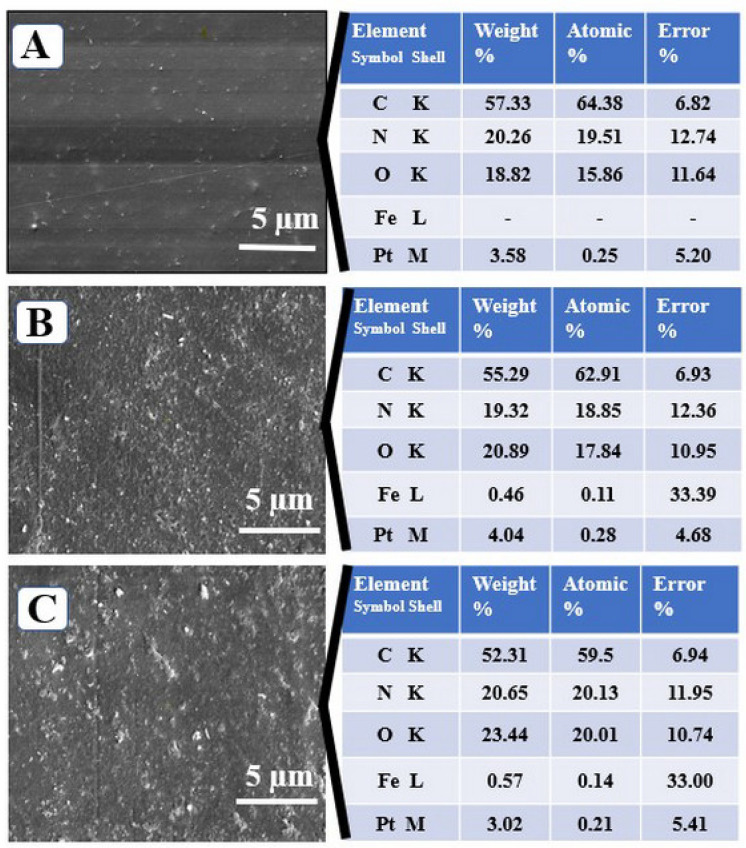
SEM micrographs of the G-material (**A**) and G-material with nanoparticles G–Fe_2_O_3_ (**B**) G–Fe_3_O_4_ (**C**) and their elemental composition.

Figure 4 pointed out the SEM micrographs of the surfaces for G and gelatin-based materials embedded with different type of iron oxides nanoparticles (G–Fe_2_O_3_ and G–Fe_3_O_4_). As it can be observed, their morphology can be attributed to the different size and granulation of nanofillers.

The SEM images of the G–Fe_2_O_3_ and G–Fe_3_O_4_ materials demonstrated that the surface of these composites is not assmooth as that of the G–material (Figure 4A).

Figure 4B shows that the Fe_2_O_3_ nanoparticles were uniformly distributed in the material microstructure, whereas Figure 4C show a slight agglomeration of the nanoparticles. This phenomenon is due to the difference in the nanoparticles size (Fe_3_O_4_ has an average size between 50 and 100 nm and Fe_2_O_3_ < 50 nm, respectively).

That behavior could be explained by the restriction of polymer chains in the presence of nanoparticles, and the arrangement of the chains indicating a rougher surface [77]. The elemental composition from the EDAX analysis confirmed once again the presence in the structure of the obtained materials of Fe_2_O_3_ and Fe_3_O_4_ nanoparticles encapsulated in the polymer matrix.

Generally, the contact angle holds the key concerning the wettability of the investigated surfaces. The parameter values were determined by using static water contact angle measurements at the materials’ surface, and then the wettability of the fabricated films was evaluated. In order to perform the measurements, the gelatin-based materials were poured on Teflon-coated slides. The determined contact angle values with water and ethylene glycol were illustrated in Table 1. The gelatin material (G sample) presents a relatively hydrophobic behavior, as is also reported in the literature [78]. This characteristic is due to the preferential orientation of the hydrophobic moieties at the gelatin–air interface during gelation; most of the hydrophilic groups (amino or carboxyl) form internal hydrogen bonds, whereas hydrophobic groups (aliphatic or aryl) arrange themselves on the surface of the film.

The gelatin contains hydrophilic chains but the contact angle measurements suggest that the film surface is relatively hydrophobic. This bipolar behavior suggests that the behavior depends on the concentration of gelatin used, so a high concentration will favor the obtainment of a film with a higher water contact angle. This high density of gelatin molecules causes a specific arrangement of molecules and the hydrophobic part of the gelatin molecules should be oriented toward the surface, whereas hydrophilic groups such as amino groups and carboxyl groups aggregate inward and form hydrogen bonds and internal hydrophilic groups [79]. The hydrophobicity of the gelatin film’s surface was determinate by the preferential orientation of the hydrophobic fragments at the gelatin–air interface [80]. The hydrated gelatin film at a higher gelatin concentration causes the orientation of apolar groups only toward air as a result of reorientation of gelatin molecules. In polyhydric phenol structure, the tannic function act as either a physical or chemical crosslinker and also bond with a multi-hydrogen function. In this strategy, the tannic function determines diverse bonding functionalities due to its five-arm polyphenol structure, enabling dense crosslinks through hydrogen and ionic bonding or hydrophobic interactions [81]. Iron ionic species form a complex or crosslink with macromolecules through multiple interactions, including hydrogen and ionic bonding, and hydrophobic interactions [82].

The results showed that the samples embedded with iron oxide nanoparticles present higher contact angle values, denoting a higher hydrophobicity compared to the sample without nanoparticles. The surface properties of the materials and the surface free energy (SFE) with its components, represent an important point to understand the laws that govern these behaviors, determining subsequent applications.

The method formulated by Owens, Wendt, Rabel, and Kaelble [83] was applied to determine the SFE with polar and dispersive contributions. The presence of polar or non-polar components on the surface of materials influences the wettability properties and induces some properties which could have an important role in applications.

The SFE values for G material and for the materials with iron oxide nanoparticles (G–Fe_2_O_3_ and G–Fe_3_O_4_) were calculated, and the results are illustrated in Table 1. The evaluation of the results indicated a considerable difference between the dispersive (γ^d^sv) and the polar component (γ^p^sv). An increase of the dispersed component can be observed from 47.65 mN/m for G material until 112.97 mN/m for G–Fe_3_O_4_. This tendency is in the same direction with the increase of the contact angle value, confirming the hydrophobic character of the sample surfaces. The value of interfacial solid–liquid tension (γ_SL_) depends on the interactions that occur between liquid and solid parts of the investigated surfaces. These values depend on the attractive forces between the molecules of the involved liquid and those of the solid surface.

### 3.3. Optical Properties

The opacity (denoted Op) parameter was determined for studied samples based on reflectance measurements on black and white supports on top of which exemplars, visible range (400–800 nm) and D5 source, are placed and is defined by the relation

Op (%) = Rb/Rw,(3)

where Rb and Rw show the sample reflectance measured on black and white supports, respectively. For the G material, it was found that Rb = 6.2 and Rw = 71.0, the ratio Op = 8.7%. For G–Fe_3_O_4_ sample 7.1%, these values are modified as follows: Rb = 4.1 and Rw = 26.3, leading to Op = 15.7%; whereas for G–Fe_2_O_3_ the values are different Rb = 6.6 and Rw = 61.6 and Op = 12.8%. Therefore, after addition of iron oxide nanoparticles in the gelatin-based-materials, the opacity was slightly increased. This increasing trend was also observed for the contrast parameter from Figure S1 [84].

### 3.4. Tensile Properties

**Figure 5:**
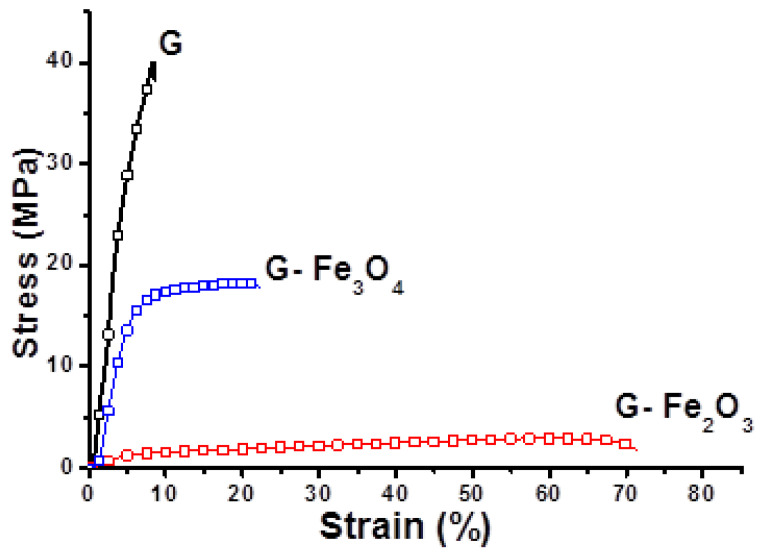
Variation of the mechanical properties at room temperature for all studied materials.

The behavior of the materials referring to the mechanical properties are determined by the configuration and architecture of the molecules, as a result of the interactions that occurs in material (Figure 5). These interactions are mostly the result of processes taking place during the orientation of macromolecules and appear after obtaining the gelatin-based films.

The mechanical properties of the materials were tracked by uniaxial tensile tests. The main mechanical properties of the materials are summarized in Table 2. Generally, the initial modulus (E) represents all the inter- and intramolecular elastic forces in the polymer matrix, which are in opposition with the polymer’s deformation. A higher value of modulus shows a more structured composite. The Young’s modulus values are listed in Table 2, and the elongation-breaking pattern has an increase for both G–Fe_3_O_4_ and G–Fe_2_O_3_.

Finally, the G–Fe_2_O_3_ material show an increased elongation compared to G material. The toughness of G–Fe_2_O_3_ material has a decrease behavior, with the stretch rates, due to the long-chain from the material.

It is possible in this case to determine a crack when the sample is stretched at a high rate, and the polymer long chains does not have time to slide until the end, and a high tension puts pressure on the short segments of the chains [85].

Many synthetic and natural materials store high strength caused by the various mechanisms. Several studies have suggested effects on crack growth in many polymers with a great porosity [86]. The mechanical properties for all samples were monitored from the stress–strain graph, from which the toughness can be evaluated. This parameter represents the amount of energy stored in the material, per volume unit, how much the material can withstand, and tensile strength indicates how much energy can be absorbed by a material before breaking itself. In other similar studies, Marvizadeh et al. [87] concluded that with the addition of iron oxide particles in the polymer system, toughness and breaking strength were decreased, and elongation was increased. The stress concentration point appears when the material subjected to an external force action will diminish the mechanical properties, producing a rupture due to those particles.

Moreover, Lee et al. [88], described an increase in toughness when ZnO particles were introduced in a similar polymer matrix. In this case, ZnO particles react with the OH groups of gelatin material, while H-bonds and covalent bonds can form the interaction between the particles and the polymer matrix, increasing the formation of crosslinks between the polymer chains. The results obtained for G–Fe_2_O_3_ and G–Fe_3_O_4_ materials can be related to the interfacial interaction, between the polymer matrix and fillers, for this behavior being responsible for the nanoparticle’s agglomeration. The increased value of the elongation at break for both materials with filler presented in Table 2, could be explained as follows: the elongation can be related to the moisture content which can have a plasticizing role in the biopolymer matrix and it can also be due to the addition of iron fillers. Thus, the decreasing of this content will decrease the flexibility of the films, which will induce a decrease in the elongation values [89,90,91].

### 3.5. Investigation of Water Vapors Sorption Capacity of Gelatin-Based Material Using the Dynanic Vapor Sorption (DVS) Measurements

Water vapors sorption/desorption capacity of the investigated gelatin-based samples was measured in dynamic regime by using a fully automated gravimetric analyzer—IGAsorp, made by Hidden Analytical (Warrington, United Kingdom). This device has an ultrasensitive balance which measures the variation of the samples’ weights as the relative humidity changes in the sample room at a constant temperature. The measurements are fully automated and controlled by a software package. After the samples were placed in a special container, they were dried at 25 °C, in a nitrogen flow, in a dynamic and continuous regime (250 mL/min) until their weights were in equilibrium at a relative humidity (RH), less than 1%. Then, the RH was gradually increased from 0 to 90%, in 10% steps, every one having a pre-established equilibrium time of 40–60 min and sorption equilibrium was obtained for every stage. After the sorption curves, the desorption curves were registered.

**Figure 6:**
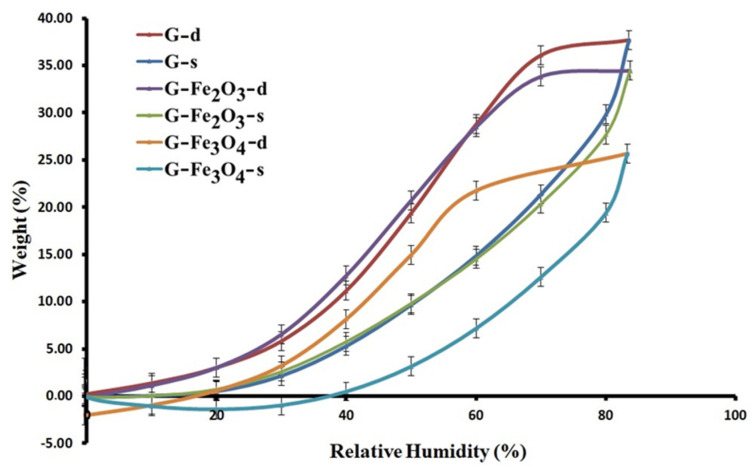
Sorption/desorption curves for the investigated gelatin-based composites.

Looking at the shape of water sorption/desorption curves presented in Figure 6, they can be associated with the type IV isotherms, according to IUPAC classification. Moreover, the type of isotherm is specific to a material with weak sorbent–water interactions. Important quantitative and qualitative information about the sorption capacity of the sample surfaces can be obtained in this way. The isotherms provided a decrease in water vapor sorption at smaller RH values (0–10%), followed by a moderate sorption at intermediary values of RH until 40% and by a significant increase of water vapor sorption at values of RH close to 100%.

The desorption curves of the isotherms for the G–Fe_3_O_4_ sample do not return to the starting point because some amount of the sorbed water in the sorption process is not desorbing and the water molecules were kept in the material matrix. The values of the surface parameters evaluated from the sorption/desorption isotherms for all the investigated samples (Figure 6) are resumed in Table 3. The results from Table 3 imply that the water absorption of the gelatin-based materials, G–Fe_3_O_4_ and G–Fe_2_O_3_ have smaller values of water vapor sorption capacity as compared with the G-material because of the presence of iron NPs, which acts as a crosslinker agent. The presence of iron Nps from gelatin-based materials induces a strengthening character. The nanoparticles in the film moves, enabling the access of the water molecules.

#### Diffusion Coefficients

According to Crank [52] and Balik [92], the diffusion coefficients were determined based on Fick’s second equation, by using the experimental data of the sorption–desorption isotherms of the three gelatin-based materials. Thus, the diffusion coefficient (*D*) can be obtained from graph, on the basis of the ratio of the polymer mass from swelling to time *t* and *t* = ∞ (corresponding to the sorption equilibrium). At short times, the *D*_1_ and K_1_ were determined from Equation (1) and at long times, the diffusion coefficients were determined by using Equation (2).

**Figure 7:**
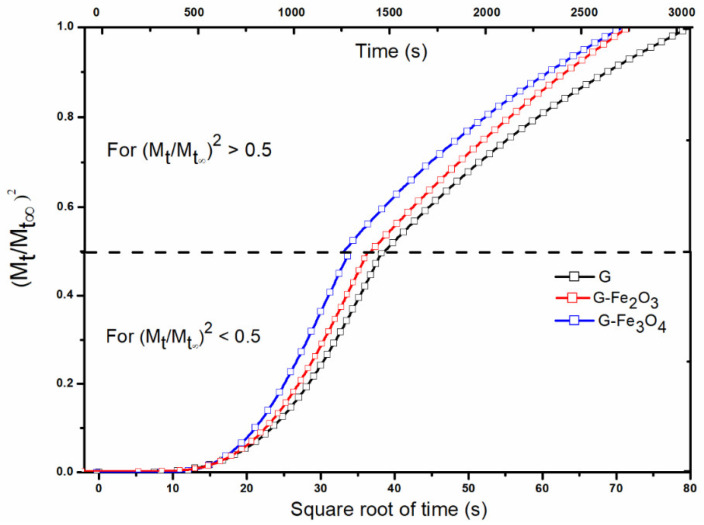
Normalized variation of the mass as function of time (G, G–Fe_2_O_3_ and G–Fe_3_O_4_).

The values of the diffusion coefficients presented lower values at the G–Fe_2_O_3_ and G–Fe_3_O_4_ materials than of the G sample (Table 4 and Figure 7). This evolution is expected because the new bonds formed between gelatin-acid tannic-iron oxide nanoparticles in the newly obtained materials will induce strong interactions. The Fe_2_O_3_ and Fe_3_O_4_ nanoparticles have a reinforcing role and stabilize the structure [93]. At long times, there is an increase in diffusion for G–Fe_3_O_4_ material compared to G control material, this phenomenon being attributed to the inhomogeneity of the material.

The slope of the graph *Mt*/*M*∞ as a quadratic function at *t*^1/2^ or the slope limit of the graph of ln (1 − *Mt*/*M*∞) are shown in Figure 7.

The larger size of Fe_3_O_4_ nanoparticles compared to Fe_2_O_3_ nanoparticles induces a decrease in cohesive force and allows the material to swell at certain points; this evolution can be caused also by agglomerations of these particles [94]. It is known that the material has both hydrophobic and hydrophilic microdomains [95].

The interfacial transport coefficient corresponds in the first phase to an interfacial resistance to the surface of the gelatin layer. The stresses that may occur generate an effect caused by elastic deformation, which associated with the adsorbed water will induce a relaxation of the material after a certain time, and the amount of water adsorbed in the system (as seen in the desorption isotherm) can no longer be removed. The diffusion coefficients clearly outline the kinetics of the process of sorption–desorption of water vapor in materials, so a decrease in these coefficients can be observed compared to G material. The low values of the diffusion coefficients are due to the obstruction of the diffusion of water vapor when the iron oxide nanoparticles embedded in the polymer matrix [96].

Another factor to consider is their size, which will allow the vapor penetration to a greater extent for G–Fe_3_O_4_ than for G–Fe_2_O_3_. The sorption–desorption isotherm for G–Fe_3_O_4_ demonstrated that some of the absorbed vapors remain trapped in the material. The diffusion coefficients increase significantly with the amount of polar groups existing in the chemical structure; in this case the hydrogen bond becomes a limiting factor in the diffusion of water vapor [97].

### 3.6. Magnetic Properties

The magnetic properties of the obtained materials are important in biomedical applications, as parts of the devices that should improve the quality of MRI investigations. The size and shape of iron oxide nanoparticles dictates the magnetic behavior of the gelatin-based samples [98,99].

**Figure 8:**
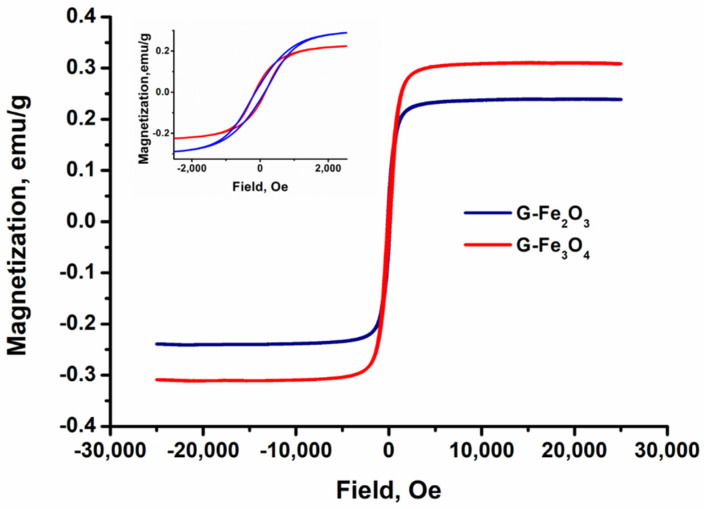
Magnetic behaviour of G–Fe_2_O_3_ and G–Fe_3_O_4_ materials at room temperature.

The saturation magnetization values of the samples from Table 5 and Figure 8 are from 5.61 emu/g for G–Fe_2_O_3_ and up to 9.38 emu/g for G–Fe_3_O_4_. It is also observed that the saturation magnetization of the magnetic samples (G–Fe_2_O_3_ and G–Fe_3_O_4_) have lower values when compared with those of the pure nanoparticles. This phenomenon is due to the surface coating of the iron oxide nanoparticles by the gelatin chains that suppress the free rotation of the magnetic moment [100].

Thus, the Fe_3_O_4_ and Fe_2_O_3_ nanoparticles, which are encapsulated in the gelatin matrix, will induce a phenomenon of attenuation of the magnetization of the nanoparticles, which is also due to the wide distribution of the dimensions of nanoparticles in the materials [101]. The small particle aggregates (for G–Fe_2_O_3_ between 100–120 nm and for G–Fe_3_O_4_ does not exceed 150) due to the interactions between the carboxylic groups and the magnetic nanoparticles and the chelating amino acids from gelatin [102,103]. In general, the saturation magnetization and coercivity values decrease as the particle size decreases [102]. The coercivity is influenced by the particle reversal mechanism and dominates the coercivity of the whole system, respectively, of the material acting as an obstacle in the spin rotation [103]. From previous reports, the interparticle dipole interaction increases with increasing NPs concentration. This interaction is more pronounced by reducing the distances between them and aggregates the coercivity of G–Fe_2_O_3_ is higher and has a lower magnetization compared to G–Fe_3_O_4_, even if the particle sizes of Fe_3_O_4_ oxide are between 50–100 nm compared to Fe_2_O_3_ with dimensions < 50 nm [104]. The magnetization curves presented a typical S-shaped superparamagnetic behavior which could improve the accuracy of MRI scans.

### 3.7. Cytocompatibility—MTT Assay

The percentage of cell viability resulting from the MTT test method is presented in order to screen the biocompatibility of the gelatin-based material without and with iron nanoparticles.

**Figure 9:**
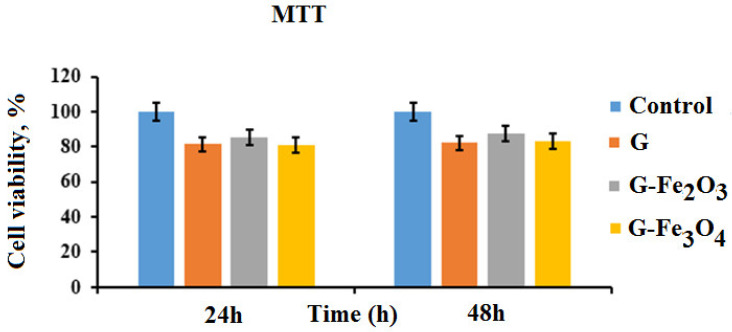
Profiles of cells grown on gelatin-based materials up to two days.

The MTT results (Figure 9) revealed that the cell viability for all gelatin-based materials with or without the iron particles were more than 80% and did not show any cytotoxic effects.

According to the values obtained in Figure 9, the magnetic nanoparticles give no toxicity in the MTT assay. All the materials with cell viability greater than 80% are often recognized as biocompatible [105]. In literature studies, some researchers uncovered iron oxide particles would be toxic [106] but those coated with polymer, as in our case, are not toxic. The materials with these embedded oxides are biocompatible, but the size of these particles is also important [107,108], due to the fact that magnetic nanoparticles can be accumulated in the target sites during a certain application.

The penetration of the particles inside the cells is highly related to their coating materials, shape, and size. Ultrasmall particles (nanospheres) can diffuse inside the cells, but the large particles cannot [109]. Figure 9 shows the results of the MTT test applied to gelatin-based materials, expressed as percentages from the viability data obtained for the control wells (i.e., the negative control). It can be observed that after 24 h of the co-incubation of the cells with the studied samples, the level of cell viability was 85.55% for G–Fe_2_O_3_, 81.39% for G material, and 80.93% for G–Fe_3_O_4_ materials. The test performed after 2 days of co-incubation showed increased levels of viability compared to a day before, for G–Fe_2_O_3_ material (87.53%) and G–Fe_3_O_4_ materials (83.19%). For G material, the increasing viability was negligible (82.07%). The differences in the cell viability observed in experimental cultures at 24 h and 48 h of material incubation are not significant, so it can be stated that the Fe_2_O_3_ and Fe_3_O_4_ nanoparticles do not express cytotoxicity for gelatin-coated constructs.

### 3.8. Antibacterial Test

The Fe_2_O_3_ nanoparticle is considered the most stable structure of iron oxides, due to its stability at various ambient conditions and pH [110,111]. The particles can be synthesized in different ways [112,113] and depending on how nanoparticles are obtained, they have many shapes and sizes [114], and their properties have a certain influence on their target destination.

The results of antibacterial activity of gelatin-based materials and iron nanoparticles were evaluated against both bacterial strains: Gram-positive (*Staphylococcus aureus*) and Gram-negative (*Escherichia coli*). The bacterial strains were selected for morpho-structural reasons, because both are representative of the bacterial groups to which they belong, are widely distributed in nature, and are frequently encountered in human and animal pathology [115,116].

**Figure 10:**
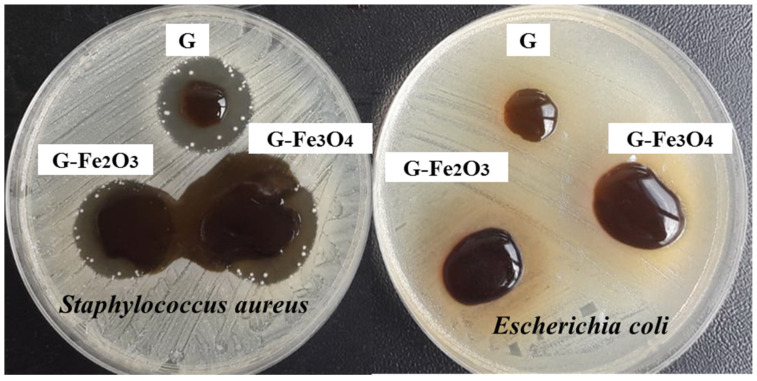
The antibacterial activity of the gelatin-based samples against *S. aureus* and *E. coli*.

A qualitative evaluation of antimicrobial activity by diffusimetric technique showed the antimicrobial potential of gelatin-based composites, but the results varied depending on the bacterial strain against which the test was performed (Figure 10).

The antimicrobial activity was interpreted by comparing the average inhibition diameters obtained after the triple test (Table 6). Because the antibiotic sensitivity of the selected reference bacterial strains is known, we chose gentamicin (10 µg) as a positive control [117].

An examination of Figure 10 showed that our gelatin-based materials did not produce areas of bacterial inhibition against *E. coli*, but a change in the volume of the samples on the surface of the medium was observed, resulting in an increase of diameter from 6 mm (original diameter) to 16 mm (G), 20.93 mm (G–Fe_2_O_3_), and 29.3 mm (G–Fe_3_O_4_). Compared to *S. aureus*, the gelatin-based materials showed the same volumetric expansion, but at the same time areas of bacterial inhibition were observed ranging from 25 mm (G), 29.3 mm (G–Fe_2_O_3_), to 38.2 mm (G–Fe_3_O_4_) (Table 6).

Comparing the zones of inhibition obtained when testing the samples with the reference zones induced by the positive control (gentamicin 10 ug), the antimicrobial effect of gelatin-based materials and iron nanoparticles is clearly highlighted (Figure S2). The dependence of the antimicrobial effect on the presence of metal nanoparticles can also be seen.

The introduction of iron nanoparticles into gelatin-based materials is a powerful tool in stabilization of materials, providing antibacterial properties [118]. The antibacterial performance of metal nanoparticles was supposed to be caused by their surface-to-volume ratio more than by the effect of metal-ion release [119]. Some studies observed that at a high surface-to-volume ratio a higher production of reactive oxygen species and free radicals was registered [120].

Thus, Fe_2_O_3_ nanoparticles proved to be effective against Gram-positive bacterial strains than Gram-negative strains.

The different behavior of the bacteria from the tested materials is due to the fact that Gram-positive bacteria have a thick cell wall (20–80 nm) consisting of a network of special structures that give the wall rigidity. In contrast, Gram-negative bacteria have a much thinner cell wall (<10 nm), but a much more complex structure that gives the cells different properties and reactions to external influences [121]. There are two possible mechanisms of action of Fe_2_O_3_ nanoparticles against Gram-positive (*S. aureus*) or Gram-negative (*E. coli*) bacteria. Even if the iron oxide nanoparticles are introduced into a matrix, they are distributed throughout the mass of the matrix. These Fe_2_O_3_ are very stable in the environment and have a lower contribution in releasing metal ions for antibacterial activity compared with other iron oxides. On the other hand, metal oxide like Fe_3_O_4_ could be a source that created ROS (reactive oxygen species) leading to the inhibition of *S. aureus*. A similar process was described by Keenan et al., in which Fe^2+^ reacted with oxygen to create hydrogen peroxide [122]. UV radiation or visible light activates the production of reactive oxygen species from G–Fe_2_O_3_ and a so-called electron hole can occur. The electron holes can contribute to the generation of ROS, such as radical anions (O^2−^), hydroxyl radicals (OH*), etc., which can lead to bacterial death [123]. Other interactions, such as electrostatics, dipole–dipole, hydrogen bonding, hydrophobicity, and Van der Waals interactions can disrupt cell function and induce or disorganize cells [124]. The absence of the antibacterial effect of gelatin-based materials with iron particles for *E. coli*, could be explained due to the rapid oxidation of the material surface. This behavior has also been found by other researchers who have studied this mechanism [125]. The antibacterial effect was also found to be a size-related and element-specific property of FeO. A substantial increase in antimicrobial activity was observed only in the absence of oxygen [126].

The gelatin-based materials induce a good antibacterial activity against *S. aureus*. The electrostatic interactions between iron oxide nanoparticles from gelatin-based material and bacterial cell membranes or cell membrane proteins can result in physical damage, and leads to bacterial cell death [127]. Such results showed that gelatin-based material embedded with iron oxides particles could have a dual function: to increase clarity when viewing images and to inhibit bacterial infection. Some researchers studied the action of the magnetic field, in which bacteria can be annihilated due to the presence of iron nanoparticles.

## 4. Conclusions

Most recent articles describe MRI materials for internal use, but this work describes a simple method by which to prepare gelatin-based materials with iron oxide nanoparticles for external use. Being in direct contact with human/animal dermis, the newly obtained materials must be accurately tuned to fit the requirements and usage conditions. The results showed that these samples embedded presented high-contact angle values, denoting a higher hydrophobicity compared to the sample without nanoparticles.

The tensile strength of the composite materials has certain values so that the film does not break during manipulation. Moreover, the differences in the cell viability observed in experimental cultures at 24 h and 48 h of incubation with materials are not significant, so it can be stated that the Fe_2_O_3_ and Fe_3_O_4_ particles do not express cytotoxicity. The gelatin-based materials induce a good antibacterial activity against *S. aureus*. Such results showed that the gelatin-based materials embedded with iron oxides nanoparticles could have a dual function: to increase clarity of images and to inhibit bacterial infection. The magnetization curves revealed a typical S-shaped superparamagnetic behavior which can improve the accuracy of MRI images. All the considerations from our study certify that these materials could be further improved or used in the current form as an external device for better clarity and accuracy of MRI images. Further work is needed to test these gelatin-based materials in MRI applications.

## Figures and Tables

**Table 1:** Surface tension parameters of gelatin-based materials with and without iron oxide nanoparticles.

Samples	Contact Angle		W_a_ (mN/m)	γ_sv_ (mN/m)	γ^p^_sv_ (mN/)	γ^d^_sv_ (mN/m)	γ_SL_ (mN/m)
	Water	Ethylene Glycol					
G	87.39 ± 0.15	45.81 ± 0.17	76.1	48.31	0.66	47.65	45.00
G–Fe_2_O_3_	115.17 ± 0.26	50.84 ± 0.21	41.83	123.36	15.02	108.33	43.60
G–Fe_3_O_4_	114.33 ± 0.19	48.15 ± 0.13	42.80	125.60	15.62	109.98	43.69

**Table 2:** Main mechanical properties for the studied samples.

Sample	Young’s Modulus (MPa) × 10^3^	Elongation at Break (%)	Tensile Strength (MPa)	Toughness (MJ/m^3^)
G	5.12	8.44	39.94	68.77
G–Fe_3_O_4_	3.35	21.88	17.95	27.15
G–Fe_2_O_3_	0.30	63.27	3.05	2.97

**Table 3:** Parameters obtained based on sorption/desorption isotherms (water vapor sorption capacity-weight-moisture content (%).

Samples	Weight (%)
G	37.70 ± 0.17
G–Fe_3_O_4_	34.46 ± 0.12
G–Fe_2_O_3_	25.79 ± 0.09

**Table 4:** The diffusion coefficients determined based on the experimental data for the analyzed samples.

Samples	K_1_*, *M_t_*/*M*_∞_ < 0.5	K_2_*, *M_t_*/*M*_∞_ > 0.5	*l* (cm)	*D*_1_ = K_1_*πl^2^*/16 (cm^2^/s)	*D*_2_ = −K_2_*l^2^*/*π^2^* (cm^2^/s)
G	4.03 × 10^−4^	−0.00156	0.1	7.91 × 10^−7^	1.58 × 10^−6^
G–Fe_3_O_4_	3.17 × 10^−4^	−0.00165	0.1	6.22 × 10^−7^	1.67 × 10^−6^
G–Fe_2_O_3_	2.95 × 10^−4^	−0.00143	0.1	5.78 × 10^−7^	1.45 × 10^−6^

**Table 5:** Magnetic properties analysis of G–Fe_2_O_3_ and G–Fe_3_O_4_.

Sample	Coercivity (Hc) (Oe)	Saturation Magnetization (Ms) emu/g
G–Fe_2_O_3_	15.07 × 10^−3^	5.61
G–Fe_3_O_4_	14.15 × 10^−3^	9.38

**Table 6:** Values of the inhibition zones (mm) for the investigated samples.

Materials	Inhibition Zone Diameter (mm)	
	*S. aureus* ATCC 25923	*E. coli* ATCC25922
G (6 mm)	25 ± 0.1155 *	16 ± 0.4619 *
G–Fe_2_O_3_ (6 mm)	29.3 ± 0.7513 *	20.93 ± 0.636 *
G–Fe_3_O_4_ (6 mm)	38.2 ± 0.8718 *	29.13 ± 0.3528 *
Gentamycin 10 ug		19–26 **

* Mean (*n* = 3); ** According to Clinical and Laboratory Standards Institute, 2009.

## Data Availability

The data that support the findings of this study are contained within the article.

## Supplementary Materials

The following supporting information can be downloaded at: https://www.mdpi.com/article/10.3390/ma15103479/s1, Figure S1: The optical photographs of the samples and Figure S2: Antimicrobial Activity of gelatin-based materials and iron nanoparticles and comparison with an antibiotic (gentamicin 10 µg).
