# Supplementary material for: Composite Materials Based on Gelatin and Iron Oxide Nanoparticles for MRI Accuracy
Source: Materials (Basel). 2022 May 12;15(10):3479. doi: 10.3390/ma15103479 (PMC9147670; doi:10.3390/ma15103479)
Supplement: Supplementary file 1 [file materials-15-03479-s001.zip › materials-1667872-supplementary.pdf]

materials

Supplementary Materials

Composite Materials Based on Gelatin and Iron Oxide Nanoparticles for MRI Accuracy

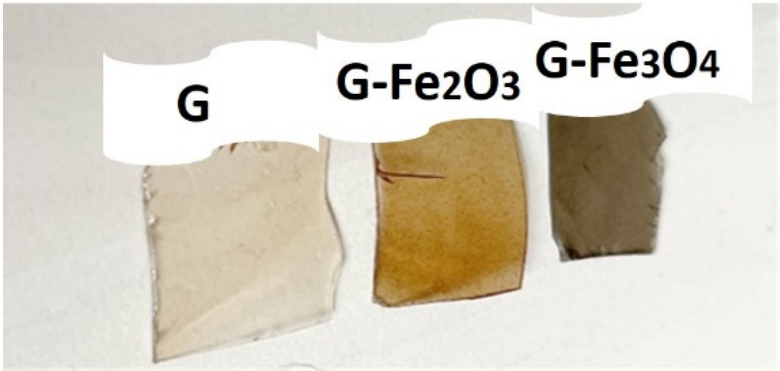

Figure S1. The optical photgraphs of the samples.

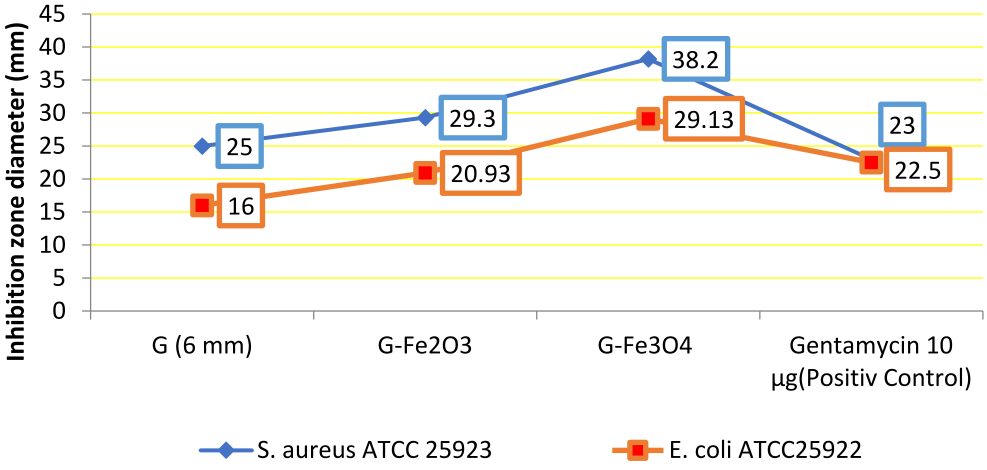

Figure S2. Antimicrobial Activity of gelatin-based materials and iron nanoparticles and comparison with an antibiotic (gentamicin 10 µg).
